# Glaucoma detection in myopic eyes using deep learning autoencoder-based regions of interest

**DOI:** 10.3389/fopht.2025.1624015

**Published:** 2025-08-04

**Authors:** Christopher Bowd, Akram Belghith, Mark Christopher, Makoto Araie, Aiko Iwase, Goji Tomita, Kyoko Ohno-Matsui, Hitomi Saito, Hiroshi Murata, Tsutomu Kikawa, Kazuhisa Sugiyama, Tomomi Higashide, Atsuya Miki, Toru Nakazawa, Makoto Aihara, Tae-Woo Kim, Christopher Kai Shun Leung, Robert N. Weinreb, Linda M. Zangwill

**Affiliations:** ^1^ Hamilton Glaucoma Center and Division of Ophthalmology Informatics and Data Science, Shiley Eye Institute, Viterbi Family Department of Ophthalmology, University of California (UC) San Diego, La Jolla, CA, United States; ^2^ Kanto Central Hospital of the Mutual Aid Association of Public School Teachers, Tokyo, Japan; ^3^ Tajimi Iwase Eye Clinic, Tajimi, Japan; ^4^ Department of Ophthalmology, Toho University Ohashi Medical Center, Tokyo, Japan; ^5^ Department of Ophthalmology and Visual Science, Tokyo Medical and Dental University, Tokyo, Japan; ^6^ Department of Ophthalmology, Graduate School of Medicine, The University of Tokyo, Tokyo, Japan; ^7^ Center Hospital of the National Center for Global Health and Medicine, Tokyo, Japan; ^8^ R&D Division, Topcon Corporation, Tokyo, Japan; ^9^ Department of Ophthalmology, Kanazawa University Graduate School of Medical Sciences, Kanazawa, Japan; ^10^ Department of Innovative Visual Science, Osaka University Graduate School of Medicine, Osaka, Japan; ^11^ Department of Myopia Control Research, Aichi Medical University Medical School, Nagakute, Japan; ^12^ Department of Ophthalmology, Tohoku University School of Medicine, Sendai, Japan; ^13^ Department of Ophthalmology, Seoul National University College of Medicine, Seoul National University Bundang Hospital, Seongnam, Republic of Korea; ^14^ Department of Ophthalmology, LKS Faculty of Medicine, The University of Hong Kong, Hong Kong, Hong Kong SAR, China

**Keywords:** glaucoma, myopia, optical coherence tomography, deep learning, artificial intelligence, diagnosis, classification

## Abstract

**Purpose:**

To evaluate the diagnostic accuracy of a deep learning autoencoder-based model utilizing regions of interest (ROI) from optical coherence tomography (OCT) texture enface images for detecting glaucoma in myopic eyes.

**Methods:**

This cross-sectional study included a total of 453 eyes from 315 participants from the multi-center "Swept-Source OCT (SS-OCT) Myopia and Glaucoma Study", composed of 268 eyes from 168 healthy individuals and 185 eyes from 147 glaucomatous individuals. All participants underwent swept-source optical coherence tomography (SS-OCT) imaging, from which texture enface images were constructed and analyzed. The study compared four methods: (1) global RNFL thickness, (2) texture enface image, (3) a single autoencoder model trained only on healthy eyes, and (4) a dual autoencoder model trained on both healthy and glaucomatous eyes. Diagnostic accuracy was assessed using the area under the receiver operating curves (AUROC) and precision recall curves (AUPRC).

**Results:**

The dual autoencoder model achieved the highest AUROC (95% CI) (0.92 [0.88, 0.95]), significantly outperforming the single autoencoder model trained only on healthy eyes (0.86 [0.83, 0.88], p = 0.01), the global RNFL thickness model (0.84 [0.80, 0.86], p = 0.003), and the texture enface model (0.83 [0.79, 0.85], p = 0.005). Using AUPRC (95% CI), the dual autoencoder model (0.86 [0.83, 0.89]) also outperformed the single autoencoder model trained only on healthy eyes (0.80 [0.78, 0.82], p = 0.02), the global RNFL thickness model (0.74 [0.70, 0.76], p = 0.001), and the texture enface model (0.71 [0.68, 0.73], p<0.001). No significant difference was observed between the global RNFL thickness measurement and the texture enface measurement (p = 0.47).

**Discussion:**

The dual autoencoder model, which integrates reconstruction errors from both healthy and glaucomatous training data, demonstrated superior diagnostic accuracy compared to the single autoencoder model, global RNFL thickness and texture enface-based approaches. These findings suggest that deep learning models leveraging ROI-based reconstruction error from texture enface images may enhance glaucoma classification in myopic eyes, providing a robust alternative to conventional structural thickness metrics.

## Introduction

Glaucoma is reportedly more prevalent in myopic eyes, particularly in highly myopic eyes (1), than in emmetropic eyes (2–4). It has been estimated that approximately 5 billion people will be affected by myopia by the year 2050. Of these, approximately 1 million will be affected by high myopia (5). Diagnosing glaucoma in myopic eyes is challenging due to structural alterations, such as optic disc tilt, peripapillary atrophy, and thinner retinal nerve fiber layers (RNFL), which complicate standard assessments such as RNFL thickness.^e.g (^ 6, 7 ^).^ Other sources of difficulties for detecting glaucoma in myopic eyes are discussed in detail by Tan et al. (8) and Jiravarnsirikul and colleaugues (9).

Recently, our group proposed texture-based enface imaging to detect glaucoma by leveraging intrinsic retinal texture properties rather than relying solely on thickness metrics (10). Results indicated that this novel texture-based analysis method can improve on standard macular ganglion cell-inner plexiform layer thickness, macular retinal nerve fiber layer thickness, and ganglion cell complex thickness maps for discriminating between highly myopic glaucomatous and highly myopic healthy eyes.

Instead of analyzing global optic nerve head (ONH) RNFL changes as is often done, focusing on specific regions of interest (ROI) may improve detection sensitivity, as glaucomatous damage tends to be localized in early stages. Bowd et al. reported that ROI-based approaches show promise in tracking glaucomatous progression by isolating regions more likely to exhibit structural deterioration (11). This method employed a single autoencoder trained on healthy eyes to detect abnormal changes based on reconstruction errors. Reconstruction errors are deviations from learned normal patterns that suggest potential glaucomatous damage. The current study expands this approach by employing two specialized autoencoders. A Healthy Autoencoder trained on normal aging eyes to model expected age-related change and a Glaucoma Autoencoder trained on glaucomatous eyes to capture disease-specific damage. This dual-autoencoder system enhances detection by comparing how well an input image aligns with learned normal or glaucomatous patterns.

The objective of the current study was to evaluate whether the dual-autoencoder framework improves glaucoma detection in myopic eyes compared to a single autoencoder model, global RNFL thickness, or texture enface image analysis alone.

## Materials and methods

For the current analysis, 453 eyes from 315 individuals participating in the multi-center "Swept-Source OCT (SS-OCT) Myopia and Glaucoma Study" (12–14) were included: 268 eyes from 168 healthy participants and 185 eyes from 147 glaucomatous participants.

Eight institutions, primarily from Asia, participated in the Swept-Source OCT (SS-OCT) Myopia and Glaucoma Study (Kanazawa University, Osaka University, Tajimi Iwase Eye Clinic, Toho University Ohashi Medical Center, Tohoku University, Seoul National University Bundang Hospital, University of California, San Diego and Hong Kong Eye Hospital). All study participants provided written informed consent according to their institution's requirements, fulfilled all inclusion and exclusion criteria, and were evaluated according to a standardized protocol. The methodology adhered to the tenets of the Declaration of Helsinki for research involving human subjects

All individuals underwent comprehensive ocular examination including refraction and corneal curvature measurements (ARK-900; NIDEK), best-corrected visual acuity (BCVA) with a 5-meter Landolt chart, axial length measurements (IOL Master; Carl Zeiss Meditec, Inc), slit-lamp biomicroscopy, Goldmann applanation tonometry, dilated fundus examination, fundus photography, stereophotography, and Humphrey Field Analyzer 24–2 Swedish Interactive Threshold Algorithm Standard testing (Carl Zeiss Meditec, Inc).

Individuals were excluded if they had a family history of glaucoma, ocular or systemic diseases that could affect VF or OCT results, or a history of systemic steroid or anti-cancer drug use. Additionally, individuals with clinically significant hypertension or hypotension (treated systolic blood pressure<100 mmHg) were excluded. All individuals were between the ages of 30 and 70 years (12, 14).

Eyes were included if they had a spherical equivalent (SE) of +1 diopter (D) or less, astigmatism<2 D, axial length<28 mm, best-corrected visual acuity (BCVA) ≥20/25, and good-quality OCT images and fundus photographs as determined by expert graders.

Eyes were excluded if there was any contraindication to pupillary dilation or if they were found to have narrow anterior chamber angles defined as Shaffer grade ≤2. Unreliable visual field results, characterized by fixation loss or false negatives >20% or false positives >15%, were also excluded. Additional exclusion criteria included the presence of optic nerve or retinal abnormalities other than glaucoma, pathologic myopia or suspected pathologic myopia (e.g., eyes with pronounced optic disc ovality >1.33 on fundus examination, inverted optic discs, posterior staphyloma, focal and/or diffuse macular chorioretinal atrophy, intrachoroidal cavitation, or circular peripapillary atrophy zones), and any history of intraocular or refractive surgery. Healthy subjects with a family history of glaucoma were excluded. Ocular or systemic conditions that could affect visual field or OCT results—such as clinically significant cataract, diabetic retinopathy, age-related macular degeneration, epiretinal membrane, or systemic steroid/anti-cancer drug use—were also grounds for exclusion, as were clinically significant hypertension or hypotension.

Normal eyes had no abnormal findings OU on complete ophthalmologic assessments including slit-lamp and fundus examinations, an intraocular pressure<21 mmHg, clinically open angles, normal optic disc appearance by stereoscopic optic disc photograph assessment (by M.A., A.I, G.T., and K.O.M.), and normal visual field (VF) results. Glaucomatous eyes were defined by the presence of a glaucomatous optic disc appearance confirmed by masked assessment of stereoscopic disc photographs (M.A., A.I., G.T., K.O.M.) and corresponding visual field (VF) defects consistent with the Hodapp–Parrish–Anderson criteria (15). Treated glaucoma patients with controlled IOP were included. All included eyes had an MD better than –12 dB, consistent with early to moderate glaucoma severity.

### Axial length-based myopia definition

We defined high axial myopia by an axial length of > 26.0 mm because axial elongation can lead to morphological changes of the optic disc and the fundus (6); myopia defined by refractive error does not necessarily reflect these changes. Moreover, cataract surgery or refractive procedures can result in a refractive shift in eyes that are axially elongated but no longer classified as (highly) myopic by refractive error. To systematically categorize axial length, we used the following classification:

- Emmetropic-Mild Myopic (<24.5 mm)
- Moderately Myopic (24.5-26.0 mm)
- Highly Myopic (>26.0 mm)

### Optical coherence tomography

All participants underwent swept-source optical coherence tomography (SS-OCT) imaging (DRI OCT Triton; Topcon, Inc). A 6.0 × 6.0 mm ONH raster scan was obtained in each eye to assess the ONH and peripapillary structures. This system uses a 1050-nm wavelength to achieve high-speed scanning and deeper tissue penetration. Each scan comprises 256 horizontal B-scans, each consisting of 512 A-scans. The scanning protocol has been previously described (12) and all OCT images deemed to meet acceptable quality standards were included in the analysis.

### Texture enface image

Texture can be characterized as a visual pattern that reflects spatial arrangement of pixel intensities of an image. Texture analysis captures the granularity and repetitive patterns of object surfaces. In the case of OCT images, each retinal layer has a unique texture that can be visually distinguished. We recently proposed a new texture descriptor called SALSA-Texture, which is robust to the intensity variation of local region caused by illumination (10). In brief, a slab 70 μm from the inner limiting membrane is created. Then for each pixel in a B-scan, a 9x9 pixel neighbouring system is created. A local normalized difference of Gaussian filter was then applied to reduce the noise and Homogeneous -bin (H-bin) normalization to increase the robustness to local contrast differences (16). In this study, the global RNFL thickness and the texture enface were both calculated using the 2–6 mm grid, where average RNFL thickness and average intensity were computed, respectively.

### Region of interest map

#### Deep learning autoencoder for glaucoma classification

Previously, we used an autoencoder to detect glaucomatous changes. For more details, see (11). Briefly, the autoencoder was trained to analyze difference images between baseline and follow-up RNFL images, identifying changes indicative of glaucoma progression. The goal of the DL-AE is to reconstruct output x′ to match the input x as closely as possible. Because the number of nodes gets progressively smaller (encoder) and then larger again (decoder) the model is forced to use the most important features of the image to be able to reconstruct the input using compressed data. The reconstruction error was used as a key indicator, with higher errors suggesting deviations from learned patterns of aging changes in glaucomatous and healthy eyes.

In this study, we expand on this approach by employing two specialized autoencoders to classify each texture enface image as either "healthy-like" or "glaucoma-like":

1. Healthy Autoencoder – Trained exclusively on texture enface images from healthy eyes, not only learning common patterns of normal ONH structure but also age-related changes and machine specific variability.
2. Glaucoma Autoencoder – Trained exclusively on texture enface images from glaucoma eyes, capturing characteristics indicative of glaucomatous damage.

Because each autoencoder is restricted to its own class, they become "specialists" in reconstructing either healthy or glaucoma-specific patterns. During inference, any texture enface image is presented to both autoencoders, generating two separate reconstructions. The reconstruction error (input map – reconstruction map) from each model provides insight into how well the image aligns with the patterns learned by each autoencoder.

The key idea is that the healthy autoencoder will reconstruct healthy-like images more accurately, leading to lower reconstruction errors for normal eyes, but higher errors when presented with glaucomatous images it has never encountered. Conversely, the glaucoma autoencoder will perform well on glaucomatous images but struggle to reconstruct healthy patterns, resulting in higher reconstruction errors for normal eyes.

Similar to our previous work, in this paper, we employed 5-layer deep learning architecture (11). We used Glorot uniform to initialize the weight matrix and the tanh activation function for the hidden layers. The model was compiled using the Adam optimization algorithm, which typically yields better results than the simpler stochastic gradient descent algorithm. To train the network, the Adam method was used to minimize the loss function (Mean Squared Error, MSE) with a learning rate of α = 10^-^³ and a batch size of 50. The deep learning autoencoder was trained end-to-end for 150 epochs, and we selected the best model based on the lowest reconstruction error.

#### Markov-based image segmentation

The reconstruction error images $\left((|\left|x-x'\right|{|}^{2}\right)$ was used to estimate the ROI map identifying areas most likely to correspond to glaucomatous structural damage. To incorporate spatial consistency and reduce noise, we applied a Markov random field (MRF)-based segmentation algorithm, which exploits the statistical correlation of reconstruction errors among neighbouring pixels (17). The MRF, a stochastic process, models the local characteristics of an image and integrates this information with observed data to infer the most probable segmentation.

Each input texture enface image was segmented into two classes, distinguishing healthy-like and glaucoma-like regions. Each pixel was represented by a 2D error feature, incorporating reconstruction errors from both healthy and glaucoma autoencoders. The MRF framework treated each pixel's classification label as a hidden variable, estimating the most probable classification while maintaining spatial smoothness in the segmentation process.

To optimize segmentation, we adopted a 2D prior-based MRF model, inspired by the approach of Kato and Pong (18) for color-textured image segmentation. In their work, multi-dimensional features—such as color and texture—were used as priors to enhance segmentation accuracy. Similarly, in our model, 2D reconstruction error features serve as priors within the MRF, allowing for more robust, spatially coherent segmentation of glaucoma-related structural damages,

#### Glaucoma classification

Instead of averaging intensity within a fixed 2–6 mm diameter region centered on the ONH using predefined concentric circles (2 mm and 6 mm), we introduce a ROI-Based Glaucoma Score that leverages the segmented region identified through MRF-based classification. This score accounts for both the extent of the affected region and the intensity reduction due to glaucomatous thinning. The ROI-Based Glaucoma Score is defined as:


$$GLS=0.5\;\text{x }\frac{ROI_{size}}{Total_{image}}+0.5\text{ x }\left(1-\frac{Mean_{ROI}}{Mean_{Healthy}}\right)$$

**Figure 1:**
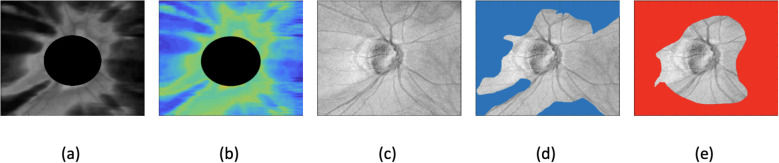
Example of glaucomatous eyes, showing **(a)** RNFL (retinal nerve fiber layer) thickness map, **(b)** texture image, **(c)** SLO (scanning laser ophthalmoscopy) image, **(d)** dual autoencoder ROI-based map, and **(e)** single autoencoder ROI-based map.

Where: 1) is the area of the segmented region identified as glaucoma-like 2).is the total image area 3) ${\text{Mean}}_{\text{ROI}}$ is the mean intensity within the glaucoma-like region and 4) ${\text{Mean}}_{\text{Healthy}}$ is the mean intensity from a training healthy dataset. **Figure 1** presents an example of a glaucomatous eye, illustrating (a) the RNFL thickness map, (b) texture image, and (c) SLO (scanning laser ophthalmoscopy) image, along with (d, e) the ROI-based maps produced by the dual and single autoencoder models.

### Training and evaluation

Five-fold cross-validation was used to provide out-of-sample predictions for auto-encoder ROI model to avoid overoptimistic estimates of classification accuracy. Both healthy and glaucoma eyes were randomly divided at the patient level into 5 subsets. For each model, we used four subsets to train the model and used the fifth subset to assess model performance. This sequence was repeated 5 times, with each subset serving as the test set one time so that each tested eye was never part of its own training set and was tested only once. An augmentation procedure in the form of horizontal mirroring similar to that used by Christopher et al. was applied to the minority class to balance the data (19).

### Statistical analyses

Descriptive statistics included the calculation of the mean and standard deviation for normally distributed variables and median, first quartile, and third quartile values for non-normally distributed variables. Student's t-tests or Mann-Whitney tests were used to evaluate the statistical significance of differences in demographic and clinical parameters between glaucoma patients and healthy individuals.

Areas under the receiver operating characteristic curves (AUROCs) and precision recall curves were used to evaluate the diagnostic accuracies of the different models investigated in the study. An AUROC regression model with maximum likelihood estimator was used to adjust for the potentially confounding effects of age, image quality, axial length, and. The difference between AUROCs was assessed using a Wald test based on the bootstrap covariance (20). In addition, precision-recall curves (AUPRCs) were computed to account for class imbalance and sensitivities at fixed specificities of 80% and 95% were calculated.

To account for the correlation between observations from the same eye, a bootstrap resampling procedure was used to derive 95% CIs and P values, where the eye-level clusters were considered as the units of resampling.

Statistical analyses were performed using Stata (StataCorp LLC, College Station, TX). P values less than 0.05 were considered statistically significant.

## Results

This cross-sectional study included 185 eyes from 147 patients diagnosed with POAG and 268 eyes from 168 healthy subjects. **Table 1** presents the demographic and clinical characteristics of the study participants and their eyes. The mean age (95% CI) at SS-OCT imaging was 51.4 (49.9, 53.0) years for the glaucoma group and 47.9 (46.3, 49.5) years for the healthy controls (p = 0.002). POAG eyes exhibited significantly worse VF mean deviation (MD) (-4.86 [-5.40, -4.32]) compared to healthy eyes (-0.31 [-0.46, -0.16]) (p< 0.001). The proportion of females was slightly higher in the glaucoma group (60.5%) compared to the healthy group (54.8%), but this difference was not statistically significant (p = 0.357).

**Table 1:** Patient and eye characteristics by diagnosis.

	Diagnosis		p-value
	Healthy (n=168, 268 Eyes)	Glaucoma (n=147, 185 Eyes)	
Age (years)	47.9 (46.3, 49.5)	51.4 (49.9, 53.0)	0.002
Sex (% Female)	54.80%	60.50%	0.25
MD (dB)	-0.31 (-0.46, -0.16)	-4.86 (-5.40, -4.32)	<0.001
IOP (mmHg)	14.28 (14.01, 14.54)	13.94 (13.61, 14.27)	0.11
AL (mm)	24.9 (24.7, 25.0)	25.6 (25.4, 25.7)	<0.001
Spherical equivalent (D)	-3.40 (-3.71, -3.09)	-4.88 (-5.26, -4.50)	<0.001
CCT (µm)	528.6 (525.0, 532.3)	530.5 (525.4, 535.6)	0.56
Corneal Radius (mm)	7.73 (7.70, 7.75)	7.74 (7.71, 7.78)	0.38

MD, Mean Deviation; IOP, Intraocular Pressure; AL, Axial Length; CCT, Central Corneal Thickness.

Mean values and 95% confidence intervals are shown for continuous variables. Statistical significance of differences in continuous and categorical variables are determined by two-sample t-tests and Fisher's exact tests for patient level variables (respectively).

Mean spherical equivalent was significantly lower in POAG eyes (-4.88 [-5.26, -4.50]) than in healthy eyes (-3.40 [-3.71, -3.09]) (p< 0.001). Additionally, axial length was significantly longer in the POAG group (25.6 [25.4, 25.7] mm) compared to the healthy group (24.9 [24.7, 25.0] mm) (p< 0.001). No significant differences were found in CCT (p = 0.565), corneal radius (p = 0.381), or IOP (p = 0.114).

AUROCs and sensitivities at 80% and 95% specificity for the single autoencoder model (trained only on healthy eyes), the dual autoencoder model (trained on both healthy and glaucomatous eyes), the global RNFL thickness measurement, and the texture enface measurement are summarized in **Table 2**. The dual autoencoder model demonstrated the highest AUROC (0.92 [0.88, 0.95]), significantly outperforming the single autoencoder model trained only on healthy eyes (0.86 [0.83, 0.88], p = 0.01), global RNFL thickness measurement (0.84 [0.80, 0.86], p = 0.003), and the texture enface measurement (0.83 [0.79, 0.85], p = 0.005).

**Table 2:** Area under the receiver operating curves (AUROC) and sensitivities at fixed specificities for each model.

	AUROC (95% CI)	80% Specificity	95% Specificity	Adjusted AUROC p-value compared to dual autoencoder ROI model
Global RNFL thickness	0.84 (0.80, 0.86)	74.3% (63.5,74.1)	64.5% (61.2,67.5)	0.003
Texture enface image	0.83 (0.79, 0.85)	71.5% (68.8,74.3)	60.1% (58.9,66.1)	0.005
Single Autoencoder ROI Model (Healthy Eyes Only)	0.86 (0.83, 0.88)	75.6% (72.5,77.3)	65.3% (63.7,69.2)	0.01
Dual Autoencoder ROI Model (Healthy & Glaucoma Eyes)	0.92 (0.88, 0.95)	81.2% (79.1,83.2)	72.4% (69.5,74.1)	NA

At 80% specificity, the dual autoencoder model achieved higher sensitivity 81.2% [79.1, 83.2], compared to 75.6% [72.5, 77.3] for the single autoencoder model trained only on healthy eyes, 74.3% [63.5, 74.1] for the global RNFL thickness measurement, and 71.5% [68.8, 74.3] for the texture enface measurement (all p-value<0.05).

At 95% specificity, the dual autoencoder model also demonstrated superior sensitivity (72.4% [69.5, 74.1]), compared to 65.3% [63.7, 69.2] for the single autoencoder model trained only on healthy eyes, 64.5% [61.2, 67.5] for the global RNFL thickness model, and 60.1% [58.9, 66.1] for the texture enface model (all p-value<0.05). There was no significant difference between the global RNFL thickness measurement and the texture enface measurement in terms of AUROC (0.84 vs. 0.83, p = 0.47).

Given the class imbalance in the dataset, AUPRC was used as it better reflects model performance in imbalanced settings by emphasizing the ability to correctly identify glaucomatous eyes while minimizing false positives. Unlike AUROC, which can be overly optimistic in imbalanced datasets, AUPRC provides a more informative evaluation of precision and recall trade-offs in this study. The dual autoencoder model achieved the highest AUPRC (0.86 [0.83, 0.89]), significantly outperforming the single autoencoder model trained only on healthy eyes (0.80 [0.78, 0.82], p = 0.02), the global RNFL thickness model (0.74 [0.70, 0.76], p = 0.001), and the texture enface model (0.71 [0.68, 0.73], p<0.001).

Because of the age difference between healthy individuals and those with glaucoma, we conducted an age-matched sub-analysis, where the age difference between healthy eyes (n = 40, mean age = 48.3 years [46.1, 50.1]) and glaucoma eyes (n = 38, mean age = 49.2 years [47.5, 51.2]) were similar (p = 0.15).

In this adjusted analysis, the dual autoencoder model (trained on both healthy and glaucomatous eyes) achieved the highest age-adjusted AUROC (0.90 [0.87, 0.93]), significantly outperforming the global RNFL thickness measurement (0.82 [0.78, 0.85], p = 0.009), the texture enface measurement (0.81 [0.77, 0.85], p = 0.004), and the single autoencoder model trained only on healthy eyes (0.85 [0.81, 0.86], p = 0.03). These results confirm that even after adjusting for age differences, the dual autoencoder model remains the most effective in distinguishing glaucoma from healthy eyes.

To assess the versatility of the dual autoencoder approach, we applied it to RNFL thickness maps instead of enface texture images. The dual autoencoder using RNFL thickness map achieved an AUROC of 0.90 [0.87, 0.93], which was similar to 0.92 [0.88, 0.95] for dual autoencoder based on using enface images (p = 0.37).

Because axial length was significantly shorter in healthy compared to glaucomatous eyes (p< 0.001), we evaluated whether variations in this measurement influenced classification performance. To address this potential confounder, we performed subgroup analyses by training the model on two AL-defined groups and testing it on the third. If a patient had one eye in the training set and the other in the test set, the training eye was removed to ensure independence between training and evaluation at the patient level. Notably, 46 eyes from 23 patients were categorized into more than one axial length groups.

**Table 3** presents the distribution of eyes by axial length category and diagnosis. Emmetropic-mild myopic eyes comprised 41.79% (112/268) of healthy eyes and 26.48% (49/185) of glaucomatous eyes (p< 0.001). The mean axial length (95% CI) in this group was 23.69 mm (23.53, 23.85) for healthy eyes and 23.79 mm (23.68, 23.89) for glaucomatous eyes (p = 0.32). Moderately myopic eyes accounted for 39.71% (105/268) of healthy eyes and 36.75% (68/185) of glaucomatous eyes (p = 0.03). The mean axial length was 26.88 mm (26.73, 27.02) in healthy eyes and 26.73 mm (26.60, 26.87) in glaucomatous eyes (p = 0.17). Highly myopic eyes represented 19.02% (51/268) of healthy eyes and 36.75% (68/185) of glaucomatous eyes (p< 0.001). The mean axial length was 25.20 mm (25.09, 25.30) in healthy eyes and 25.27 mm (25.18, 25.36) in glaucomatous eyes (p = 0.57).

**Table 3:** Distribution of eyes by axial length (AL) category and diagnosis.

		Diagnosis		p-value
		Healthy (n=168, 268 Eyes)	Glaucoma (n=147, 185 Eyes)	
Emmetropic-mild myopic eyes	Number of eyes	112	49	
	% of total	41.8%	26.5	<0.001
	AL (mm)	23.7 (23.5, 23.8)	23.8 (23.6, 23.9)	0.32
Moderately myopic eyes	Number of eyes	105	68	
	%	39.7%	36.7%	0.03
	AL (mm)	26.9 (26.7, 27.0)	26.7 (26.6, 26.9)	0.17
Highly myopic eyes	Number of eyes	51	68	
	% of total	19.0%	36.7%	<0.001
	AL (mm)	25.20(25.1, 25.3)	25.3 (25.2, 25.4)	0.57

Percentage distributions and mean AL values with 95% confidence intervals are shown for each category. Statistical significance of differences between healthy and glaucoma groups is determined by chi-square tests for categorical distributions and two-sample t-tests for AL comparisons.

For emmetropic-mild myopic eyes, the dual autoencoder model (trained on two other groups) achieved an AUROC of 0.92 [0.89, 0.94], significantly outperforming the single autoencoder model (0.86 [0.81, 0.88], p = 0.03). For moderately myopic eyes, the dual autoencoder model trained on two other groups achieved an AUROC of 0.90 [0.86, 0.92], outperforming the single autoencoder model (0.83 [0.79, 0.85], p = 0.01). For highly myopic eyes, the dual autoencoder model trained on two other groups achieved an AUROC of 0.88 [0.85, 0.91], outperforming the single autoencoder model (0.80 [0.77, 0.83], p = 0.004).

## Discussion

The results of the current study indicate that dual autoencoder trained on normal aging eyes to model expected age-related change and trained on glaucomatous eyes to capture disease-specific damage achieved the highest AUROCs significantly outperforming the single autoencoder, global RNFL thickness, and texture enface models with p< 0.01. The glaucoma-trained autoencoder accounted for disease-specific changes beyond normal aging, improving classification accuracy, particularly in myopic eyes, where normal variations can obscure glaucomatous damage.

In myopic eyes, elongated axial length alters optic nerve morphology, making traditional optic nerve head based diagnostic metrics less reliable as previously discussed. By incorporating both normal aging and glaucomatous damage, the dual autoencoder differentiates between natural variations and pathology more effectively than a model trained only on healthy eyes. Further, the ROI-based method allows for targeted analysis of regions of the ONH most susceptible to glaucomatous damage, reducing false negatives, as shown previously. From a clinical standpoint, the region of interest-based, combined healthy autoencoder- and glaucoma autoencoder-trained dual autoencoder approach could complement standard thickness measurements or replace them in cases where conventional RNFL metrics fail to provide clear diagnostic insights.

Other studies have investigated the use of texture analysis for detecting glaucoma and myopia with varying degrees of success. Leung and colleagues introduced RNFL Optical Texture Analysis (ROTA) to reveal the optical texture and trajectories of individual axonal fiber bundles including the optic nerve head region and the macula (21). This method integrates both RNFL thickness and RNFL reflectance measurements obtained from standard OCT scans. Results indicated that papillofoveal and papillomacular bundle defects previously thought to develop late in the disease were common in early glaucoma. In addition, ROTA defined defects significantly increased the likelihood of abnormality in the corresponding central visual field defects (Humphrey Field Analyzer 10–2 protocol) (22). With regards to myopia, the texture-based assessment PAMELA (Pathological Myopia Detection Through Peripapillary Atrophy) has been shown to automatically assess a retinal fundus image for pathological myopia using an SVM classification (23). In addition, a sensitivity of 0.90 and a specificity of 0.94 with a total accuracy of up to 92.5% was obtained for detecting pathological myopia using a modified version of PAMELA incorporating grey level analysis (24). Finally, In an earlier study, Bowd et al. (10) reported that the performance of SALSA-texture analysis used in the current study for differentiating between healthy and highly myopic glaucoma eyes was superior to that reported currently (AUROCs 0.92; 95% CI: 0.88-0.94 versus 0.83; 95% CI: 0.79-0.85). The inferior performance of texture en face images in the present study may be attributed to differences in subject demographics and characteristics.

Machine learning analysis of OCT measurements for detecting glaucoma in myopic eyes also has been reported. For instance, in one such study by Kim et al. (25) several convolutional neural network-based DL models were compared for classifying highly myopic healthy and highly myopic glaucomatous eyes with large PPA areas affecting circumpapillary OCT scans. In their study, an EfficientNet-B0 model trained and tested on macular vertical OCT measurements outperformed a model trained and tested on circumpapillary OCT measurements with AUROCs of 0.981 (0.955-1.00) and 0.840 (0.769, 0.912), respectively. Significant differences using other models (DenseNet-121, VGG-13, ResNet-34, ResNet-101, EfficientNet-B1) were not observed.

There are several strengths to using the novel SALSA-texture auto-encoder based detection of glaucoma using OCT ONH images. First, SALSA-texture auto-encoder analysis can achieve high diagnostic accuracy, both AUROC and AUPRC, with a relatively small sample size. In addition, in contrast to ROTA (21) which requires segmentation of both the ILM and RNFL layers, SALSA-Texture requires segmentation of only 1 layer, as it is applied to tissue in a 70 micron slab below the ILM. Requiring fewer segmented ONH layers reduces the likelihood of segmentation errors which can lead to artifacts and erroneous results. Finally, the use of a dual autoencoder trained on both healthy aging eyes to model expected age-related change and glaucomatous eyes to capture disease-specific damage resulted in an improvement in glaucoma detection performance compared to a single autoencoder trained on healthy eyes alone.

The current study is not without limitations. First, the available dataset is rather small. Although the model demonstrated strong performance with a relatively small sample size, larger datasets are needed to validate its robustness. Because of the small sample size, we relied on cross-validation instead of an external dataset to test model accuracy. However, an external dataset is preferrable so that the generalizability of the models can be evaluated, and to reduce the likelihood of overfitting. The reported findings need replication across different populations and imaging systems.

The population in the current study was largely Asian, with 5 Japanese sites, 1 Korean site, 1 Chinese site and 1 U.S. site limiting generalizability of results across races. Finally, training was instrument specific. The model was trained on a single OCT device, and performance across other imaging systems remains untested. To overcome these limitations, future directions include dataset expansion, cross-validation and integration with functional testing. Ideally, we would 1) validate the model on a multi-center, larger, and more diverse cohort to ensure generalizability; 2) test performance using different OCT devices and imaging protocols to enhance applicability, and 3) combine autoencoder-based structural analysis with visual field testing to create a more comprehensive glaucoma detection framework.

Another limitation of this study is the use of RNFL thickness as the sole structural comparator for evaluating the performance of our texture-based autoencoder model. Although RNFL is commonly used in clinical glaucoma assessment, macular ganglion cell complex (GCC) parameters have shown strong diagnostic utility in highly myopic eyes. Notably, prior studies (26, 27) have reported that GCC and GCIPL metrics are often less affected by optic disc tilt and peripapillary atrophy than RNFL measures in this population. We selected a texture-based approach specifically because it requires only ILM segmentation, which tends to remain reliable even in the presence of myopia-related structural distortion—unlike segmentation of deeper layers around the ONH. Since the 6×6 mm scan used in this study captures both ONH and macular regions, future research will investigate the added value of incorporating GCC thickness as a complementary structural comparator to texture and RNFL features.

Moreover, further improvements in our ROI-based framework could be realized by integrating complementary biomarkers beyond structural texture. For example, OCT angiography-derived vessel density has been shown to decline in glaucomatous eyes with high myopia and may provide vascular insight where structural metrics are ambiguous (28). In addition, retinal ganglion cell metabolic dysfunction, especially involving mitochondrial pathways, plays a critical role in glaucomatous neurodegeneration. Imaging methods such as flavoprotein autofluorescence have been used to noninvasively assess mitochondrial activity *in vivo* and could offer valuable functional context to structural findings (29). Incorporating such multimodal features into future versions of our dual-autoencoder model may further optimize glaucoma detection in myopic eyes.

Another limitation of this study is that the model was developed and evaluated solely on emmetropic to myopic eyes. While this was appropriate given the study's objective to improve glaucoma detection in myopia—a group where diagnostic performance is often suboptimal—it may limit generalizability to other refractive categories such as hyperopia. Hyperopic eyes can exhibit distinct optic nerve head morphologies and retinal structures, which may influence model performance. Future validation in more diverse refractive populations, including hyperopic and astigmatic eyes, will be essential to assess the broader clinical applicability of the proposed approach.

The ROI-Based Glaucoma Score generated by the dual autoencoder framework represents the extent and severity of localized deviations in retinal texture, which may serve as a quantitative indicator of glaucomatous damage. To support clinical translation, future efforts will focus on building a normative database to establish reference ranges and confidence intervals for the score enabling percentile-based interpretation. This will allow clinicians to contextualize patient scores within a population distribution, facilitating risk stratification and decision support. Additionally, prospective validation will be necessary to determine score thresholds for diagnostic or monitoring use in clinical workflows.

In conclusion, the dual autoencoder ROI-based approach significantly improves glaucoma detection in myopic eyes by accounting for both normal aging and disease-specific damage. This method shows promise for improving our ability to utilize optic nerve head OCT images to detect glaucoma in myopic eyes.

## Data Availability

The raw data supporting the conclusions of this article will be made available by the authors, without undue reservation.
